# Multi-Criterial Analysis Tool to Design a Hybrid Ballistic Plate

**DOI:** 10.3390/ma14144058

**Published:** 2021-07-20

**Authors:** Marcin H. Struszczyk, Paulina Dmowska-Jasek, Paweł Kubiak, Marcin Łandwijt, Marzena Fejdyś

**Affiliations:** Institute of Security Technologies “MORATEX”, Marii Sklodowskiej-Curie 3 Street, 90-505 Lodz, Poland; mstruszczyk@moratex.eu (M.H.S.); pkubiak@moratex.eu (P.K.); mlandwijt@moratex.eu (M.Ł.); mfejdys@moratex.eu (M.F.)

**Keywords:** hybrid ballistic plates, one-step forming process, multi-criterial analysis, ballistic product design, ballistic protection, ballistic materials

## Abstract

The presented research focuses on the concept of an advanced ballistic personal protection design, taking into account safety as well as performance requirements. The application of the multi-criterial analysis (MCA) allows for a comprehensive comparison of all the properties of materials and to select the optimal personal ballistic protection system, considering their mechanical and ballistic properties. The newly developed hybrid ballistic composites, consisting of two or three various components (variations of ballistic and/or non-ballistic textiles; hybrid ballistic plates—HBP), were evaluated via a multi-criterial analysis that considered a wide range of properties, describing behavior and safety usage, as well as the economical aspect of their fabrication.

## 1. Introduction

Body armor (e.g., ballistic plates) is a piece of individual protection equipment that is designed to protect the wearer against small-caliber ammunitions by reducing or even preventing projectile penetration into the human body and dispersing projectile impact energy [1].

Selection of the raw material when designing ballistic personal protection (BPP) is the most crucial for achieving the required properties of the final product. Several parameters describe the BPP behavior such as ballistic resistance, mass, area of protection, and additional functionalities (e.g., spike, and knife resistance). Moreover, several other features define the BPP properties, which could be difficult to compare (such as the cost of the main ballistic components, comfort aspects, usability, etc.) with the above-mentioned properties, also between each design. A wide range of different types of textile materials used, e.g., in ballistic products, and their physical and mechanical properties were recently described [2,3]. Also, the design history, methods, ergonomics, and usability aspects were widely discussed.

In the presented article, the most critical properties for selecting the optimal group of BPP features were as follows: ballistic resistance (including projectile velocity, V50), usage safety (including depression depth, area of depression, depression volume, and protection area), mechanical properties (mechanical strength, tear resistance, and hardness), physical properties (areal density, and thickness), stability (thermal stability, and time stability), ergonomic behavior, and economic aspects.

Designing body armor, made with different ballistic and non-ballistic materials faces several crucial problems and consequences for the users. Researchers and engineers are trying to solve various user-perspective cases, such as product heaviness, long-time fatigue, or to maximize the ballistic protection area. These problems often result in mobility issues, and higher fatigue or stress in real usage [4]. There is an urgent need to improve the ballistic product’s design and manufacture, to help solve crucial physical and psychological aspects for users, as well as the final product costs and manufacturing repeatability.

A useful tool for proper selection of the optimal solution for BPP design is the multi-criterial analysis (MCA), proposed by Zurek [5] and Karolinski [6]. Adoption of the MCA was performed by Struszczyk [7,8] for selecting the most optimal variant of hernia implants made by knitting.

The main idea of using textile materials for manufacturing composite ballistics plates is to increase their durability while reducing their mass. Moreover, the plates should ensure ideal fitting to the user’s body, further increasing the comfort of usage and preserving their bulletproof properties. The ballistic resistance of composite ballistic plates varies widely and depends primarily on the characteristics of the selected material, by the design of the product, and manufacturing technology [9]. Composite ballistic plates are fabricated using fiber-based pressing technology enabling anatomically contoured structures to be shaped in a single technological operation if one type of textile is used. However, application of two or more types of ballistic textiles, the molding temperature of which differs, requires more than one stage in the process [10].

Ballistic textiles are one of the main groups of materials, used widely in ballistic protection systems. Nowadays, a lot of research has described the wide range of ballistic properties (such as the V50 parameter or backing material deformation [11,12]) for various materials, to help define the best components that could be used for maximizing the ballistic performance of various protection systems. While developing ballistic products, various non-ballistic materials are used to help provide an adequate level of protection, and to improve its mechanical properties. In some cases, using non-ballistic materials in addition to manufacturing the ballistic plates could also improve its ballistic properties [13] as well as the weight reduction of the final product. Complex research on the ballistic performance of lightweight hybrid composites, used for body armor was described by Yanen and Solmaz [14]. In ballistic protective products, even if the projectile penetration may be prevented, serious and even lethal injuries can be caused by the armor-released high-impact energy transferred to the body. This injury type, caused by deformation or energy transmission into the body during ballistic impacts, even without visible bullet penetration, is called: Behind Armor Blunt Trauma (BABT) [15].

In this article, various materials as part of the ballistic product structure were described. For example, the woven kenaf p-aramid (Kevlar^®^) hybrid composites were fabricated and tested in [16,17] using fragment-simulating projectiles at various impacts as well as velocities. The hybrid composites were characterized by their required ballistic performance. The thickness and areal affected the ballistic behavior of the hybrid composites. The performance of the composite designed by Kevlar^®^ 29 fabrics impregnated with thermosetting resin was evaluated by Sorrentino [18]. The fabricated ballistic systems were analyzed by the V50 parameter to show the suitability of the performed process of fabrication and selection of the materials. Also, the ballistic performance of the UHMWPE panels with different thicknesses were tested by fragment and characterized by back-face deformation using a spherical projectile [19]. The above-mentioned parameters were used for analyzing the influence of the fiber orientation as well as for analyzing limitations on the deformation and failure response to the ballistic loading conditions. According to Crouch [20], there is a new trend with ultra-high molecular weight polyethylene fibers that enables new designs to evolve in new ballistic systems.

The aim of the present research was to adopt hybrid ballistic plates (HBP) based on the MCA as well as to perform a selection of the optimal variant of ballistic inserts for the bullet- and fragment-proof vests. The HBPs were fabricated in a one-stage pressing process, allowing for a combination of a variety of textiles, both ballistic and non-ballistic, varying in their molding temperatures. The MCA process was helpful for the selection of the proper raw materials, and the design of the HBP originating from the originally designed one-stage fabrication process.

## 2. Materials and Methods

### 2.1. Materials—Ballistic Materials

Three kinds of soft fibrous composites consisting of the UHMWPE fibers manufactured by DSM High Performance Fibers BV/The Netherlands, were described and used to make the hybrid ballistic plates (HBP). The HBP also consisted of non-impregnated Twaron^®^ CT736 p-aramid woven fabric (Tenjin/The Netherlands). The material properties are presented in Table 1.

### 2.2. Materials—Non-Ballistic Materials

When producing HBP, which contains non-impregnated p-aramid fabric as one of the ballistic materials, it was necessary to use a suitable binder. The choice of thermoadhesive film was extremely important due to the need to reduce the standard pressing temperature of p-aramid woven fabrics from 175 to 128 °C. Thermoadhesive film of type “I” (Supplier: AGAWA.PL Ltd., Tomaszow Mazowiecki, Poland) with a thickness of 73 μm and an area density of 80 g/m^2^ was selected.

A non-ballistic textile, in the form of the non-woven product: Nonwovens for Fiber Reinforced Plastics T1790 C (GRM Systems, Olomouc, Czech Republic) was used to fabricate a hybrid, three-component HBP to minimize deformation caused by the projectile impact, and consequently, to reduce the “ballistic trauma” effect—BABT.

Another non-ballistic textile in the form of a woven carbon fabric—Carbon Fabric CBXS 400 (GRM Systems, Czech Republic)—was additionally applied to fabricate a hybrid, three-component HBP, also to minimize the deformation from the projectile impact and to reduce the BABT effect. The main properties of the applied materials: Reinforced Plastics T1790 C CBXS 400 fabric are shown in Table 2.

### 2.3. Methods 

#### 2.3.1. One-Stage Process of HBP Fabrication

The one-step HBP forming process was carried out by pressing the UHMWPE fibrous composites under a PHM-2000E press (Ponar Zywiec, Zywiec, Poland) using a flat mold measuring 25 × 30 cm. The above-mentioned process was carried out according to the patent applications [21,22].

The process parameters, described below, contain different durations of the various pressing stages:

- pre-pressing stage: 45 min at temperatures from 20 to 60 °C up to 128 °C under a pressure of 10 bar;
- main pressing stage: 40 min at a temperature of 128 °C under a pressure of 200 bar;
- cooling stage: 45 min until reaching a temperature of 60 °C.

HBP are made of ballistic and/or non-ballistic materials based on:

- soft UHMWPE fibrous composites: Dyneema^®^ HB212, HB210, or HB26;
- non-impregnated, p-aramid woven fabric: Twaron^®^ CT736;
- connecting binder: thermoadhesive film, which is the connecting layer of the aforementioned ballistic materials, and one of the following non-ballistic textiles: nonwoven glass fiber: Nonwoven Fiber Reinforced Plastics T1780 C or carbon woven fabric: Carbon Fabric CBXS 400.

Table 3 presents the characteristics of the HBP variants.

Table 4 presents a list of materials used to fabricate the HBP (three-components hybrid). The HBP was made in two variants with different material configurations, although the quantity of material layers remained the same in both cases:

- Variant A: arrangement of material layers in the direction from the user’s body is as follows:

non-impregnated, p-aramid woven fabric: Twaron^®^ CT736/connecting binder - thermoadhesive film nonwoven glass fiber: Nonwovens for Fiber Reinforced Plastics T1780 C or carbon woven fabric: Carbon Fabric CBXS 400 → soft UHMWPE fibrous composite: Dyneema^®^ HB212.

- Variant B: arrangement of the material layers in the direction from the user’s body is as follows:

non-impregnated, p-aramid woven fabric: Twaron^®^ CT736/connecting binder - thermoadhesive film → soft UHMWPE fibrous composite: Dyneema^®^ HB212 → nonwoven glass fiber: Nonwovens for Fiber Reinforced Plastics T1780 C or carbon woven fabric: Carbon Fabric CBXS 400.

#### 2.3.2. Areal Density

The areal density of the HBP was determined using the following equation:

D = (m × 10 ^6^)/A [g/m^2^]; (1)

where:

m—weight of sample [g];

A—surface area of sample [mm^2^].

#### 2.3.3. Thickness

The thickness of the HBP was determined as the distance between the top and the bottom of the sample, under a preliminary pressure of 2.0 kPa ± 0.2 kPa. The sample was positioned for 10 s between a round pressing foot with a smooth flat surface (with a diameter of 9 mm) and a thickness gauge table (50 mm in diameter, larger than the diameter of the pressing foot).

#### 2.3.4. Ballistic Properties

**Figure 1:**
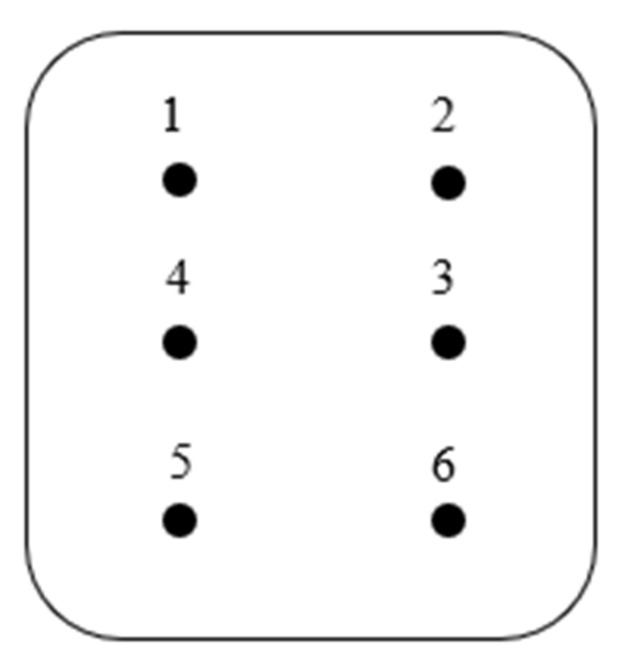
Shot sequence: 1 ÷ 6 shots at an angle of 0 ± 5°.

The bullet resistance determination of the fabricated HBP was carried out using the 7.62 mm PS FMJ projectile (authors internal procedure, based on the normative document PN-V-87000:2011, Class K3A): 6 shots were fired (at an angle of 0 ± 5°) in the projectile velocity range: 350 ÷ 735 m/s. The samples were tested in a dry state under ambient temperature conditions 20 ± 5 °C, at a relative humidity of 65 ± 10 %. The shot sequence is shown in Figure 1.

In addition, for each impact of the projectile, the volume of the substrate deformation (*V_dp_*) and the pattern of the substrate deformation field (*P_dp_*) were determined based on the following equations:

(2) $$V_{dp}=\frac{2}{3}\times \pi \times a\times b\times c\;[{\mathrm{cm}}^{3}];$$

(3) $$P_{dp}=\pi \times a\times b\;[{\mathrm{cm}}^{2}];$$

where:

*a*, *b*—semi-major and semi-minor axes of the ellipse;

*c*—depth of deformation of the substrate.

#### 2.3.5. Multi-Criterial Analysis (MCA)

Selection of the configuration for the HBP design was performed based on the results of the multi-criterial analysis (MCA), which was determined by the General Coefficient of Quality (GCQ), using relative, parameterized criteria for each group of parameters determining a characteristic or functional property being critical to the selection of the optimal variant.

The following parameters (aggregated into two groups) were applied in the MCA:

(1) physical properties (areal density, and thickness);
(2) usable properties (average projectile velocity, maximum depth of depression, area of deformation, volume of deformation, and ballistic penetration).

Criterial markers (x_i_) were selected with regards to the parameters describing the safety and performance of the designed variants of the HBP models. The above-mentioned coefficients were calculated according to [5,6,7,8,9]:

x_i_ = ( k_i_ − k_min_ )/(k_max_ − k_min_) (4)

if, as the value increases, characteristics of the HBP are improved

x_i_ = 1 − (k_i_ − k_min_)/(k_max −_ k_min_) (5)

if, as the value increases, characteristics deteriorate the performance of the HBP.

The k_min_ and k_max_ values were determined based on the testing parameters. For the two-components HBP the whole range of the parameters of these HBP were used for the determination of the k_min_ and k_max_, whereas for the three-components HBP the parameters of the two- and three-components HBP were applied.

Sectional Coefficients of Quality (SCQ) and GCQ were calculated according to [5,6]. For criterial markers describing the characteristics, Sectional Coefficients of Quality (SCQ) were calculated according to the formula:

$$SCQ_{i}=\sum_{i=1}^{n}x_{i}\times \alpha_{i},$$

where:

*x_i_*—criterial marker for a given feature;

*α_i_*—correction factors depending on the degree of importance of *t_i_* (validity) for the feature according to the formula:

$$\alpha_{i}=\frac{t_{i}}{\sum_{i=1}^{n}t_{i}},$$

In this study, the validities presented in Table 5 were used for the MCA realization.

The General Coefficients of Quality (GCQ) were calculated according to the equation:

$$GCQ=\frac{1}{n}\sum_{i=1}^{n}SCQ_{i},$$

The calculated quality indicators have been classified in the relevant quality class based on the formula:

$$K=10-\left(10N+u\right),$$

where: 

*K*—quality class (0 is excellent and 9 is very unfavorable);

*N*—value of the quality indicator SCQi or GCQ;

*u*—decimal fraction supplementing the product 10 × N to the nearest whole number.

Generally, GCQ and SCQ ranges are estimated to be between 0 and 1, where 1 indicates the ideal quality and 0 indicates the worst quality.

The quality coefficients were classified into an appropriate quality class (C), where C = 0: ideal, and C = 9: most unfavorable [5,6].

## 3. Results and Discussion

In the first stage of the research, various HBP were preliminary ballistic tests. The considered HBP were made with various ballistic textiles such as: Twaron^®^ CT736 and Dyneema^®^ HB26, HB210, or HB212 as well as with layers of glass fiber nonwovens (indicated with S in the HBP code) and a carbon woven fiber layer (with W in their code). Moreover, they have been fabricated in two variants (indicated as A or B) with various layer configurations at the body side. At this stage, they were tested in a wide bullet speed range (from 300 up to 800 m/s) and reported with an overshot in some cases (for example H1/S failed at multiple shots with different speeds, as well as H4/SB and H4/WB with the shot at >715 m/s).

Table 6 presents the determined research parameters characterizing the designed HBP, of both the two- and three-components.

The presented data characterized several features of the fabricated HBP that can hardly be compared between each other. The selection of the optimal HBP variants is difficult without the appropriate analytical tools dedicated to them.

Table 7 presents the selection of the optimal two-components HBP variants.

Initially, the selection of the optimal variants of the two-components of the HBP based on the three-components of the HBP was performed. The highest SCQ_Ph_ was found in the case of the fabricated HBP using Dyneema^®^ HB212 and Dyneema^®^ HB210 fibrous composite (C_Ph_ = 0). Moreover, the number reduction of the p-aramid woven fabric in the HBP yielded an increase in SCQ_Ph_ as shown for the H1 ÷ H3 and H4 ÷ H6 variants. The worst quality was found for the HBP partially made of the Dyneema^®^ HB26 fibrous composite (C_Ph_ ranging from 9 to 8). Patrial exchange of the Dyneema^®^ HB26 by Dyneema^®^ HB212 resulted in a significant improvement in the quality (H10; SCQ_Ph_ = 0.79). The best quality of HBP in respect to usable properties for HBP was made of the Dyneema^®^ HB26 and Dyneema^®^ HB212 fibrous composite (C_U_ ranging from 4 to 3), whereas the HBP containing the Dyneema^®^ HB210 fibrous composite was characterized by the lowest quality class (H1 and H2). Generally, the better overall quality was found in the HBP fabricated in participation with Dyneema^®^ HB212 (H4–H6; Quality Class = 2; GCQ ranging from 0.71 to 0.80). The above-mentioned configurations were chosen for the next stage of the research: designing the three-components HBP. H4 samples are characterized by their ballistic penetration, so their selection resulted in the use of the whole range of the material configurations of HBP for the verification of the influence of the implementation of the third component for the ballistic performance.

Additionally, implementation of the non-ballistic materials in the H4 configuration (three-components system) resulted in ballistic penetration, whereas the H5 and H6 configurations with the addition of the above-mentioned textiles were characterized by a positive ballistic performance—no penetration (Table 5). The SCQs of the three-components HBP showed a slight reduction in quality as compared with the two-components HBP (Table 8). The best C_Ph_ was found for the H4/SB and H6/SB samples (external implementation of the glass fiber containing nonwoven in the composite), whereas HBP containing the carbon woven fabric was characterized by the worst C_Ph_.

In most cases, the C_U_ of the HBP three-components was worse compared with the quality of the two-components HBP. The internal implementation of the carbon fabric (with the reduction of the p-aramid woven fabric participation) or external implementation of the glass fiber nonwoven gave a better quality (C_U_ = 5) than the other HBP three-components. Moreover, the reduction in the p-aramid woven fabric in parallel with the external application of the carbon woven fabric yielded a C_U_ ranging from 5 to 4 (better quality than for the similar configuration with the highest amount of p-aramid woven fabric). The above phenomenon is connected with the stiffening of the composite structure supporting the preferable expansion of the bullet energy on the higher surface of the composite.

Mostly, the reduction of the p-aramid woven fabric amount in the three-components HBP yielded the improvement of the quality of the ballistic composite (GQC = 3). Internal application of the glass fiber non-woven with reductions in the p-aramid woven fabrics showed an improvement of the GQC (GQC = 2).

Various kinds of ballistic plate variants have been tested in the first stages of the HBP research. The considered HBP were made with various ballistic materials, such as: Twaron^®^ CT736 and Dyneema^®^ HB26, HB210, or HB212 as well as with layers of fiberglass and carbon. Despite the HBP structure, the crucial parameter when choosing the proper variants for further development was a ballistic penetration test. According to the ballistic results, some variants with higher GCQ failed in the ballistic penetration test (i.e., the H4 configuration of HBP), resulting in an overshoot. Due to user safety, these variants did not provide the required ballistic protection, so they cannot be chosen for further research.

The most optimal HBP variants (with the ballistic performance level aspect) for further development have been chosen as follows:

(a) two-components: H5—H6 configurations; GCQ = 0.80;
(b) three-components: H5/SA and H6/SA; GCQ = 0.75.

The selected HBP that showed optimal performance and safety was characterized by:

- in the two-components group: the lowest thickness correlated with low areal density and low depth of the deformation, as well as deformation area and volume corresponding with an increase in the safety of the ballistic protecting the users. The safety of the selected HBP was also confirmed by no penetration during the ballistic tests;
- in the three-components group: low thickness and low areal density with relatively low depth of the deformation and deformation area as well as volume resulted in no penetration during the ballistic test.

## 4. Conclusions

Using the MCA, grouped parameters describing the performance and safety of the newly developed HBP variants were converted to criterial markers. The applied parameters have been described by various units, and therefore, it is convenient to use relative coefficients to describe the particular features of the ballistic composites. With the applied adaptation of the MCA, it is possible to choose the best quality variants of HBP and describe the mechanism of the ballistic taking into account the physical behavior of the HBP with various configurations. In our overall evaluation of the two- and three-components HBP, those containing only ballistic textiles showed the best usable behavior. Otherwise, the application of the non-ballistic textiles implemented additional value (i.e., stiffening of the composites) that could influence a single feature, but did not affect the overall performance of the newly developed HBP. The ease of adopting the MCA has several advantages in the design of the technology accounting for the performance and safety of the resulting ballistic plates, particularly when the configuration of the composites and the number of features being analyzed are widely expanded.

Due to the parametrically grouped characteristics of the HBP variants and comparing them based on various functional characteristics with various names, providing a validated tool is crucial to choose the best variant with the required qualities.

## Figures and Tables

**Table 1:** Properties of the soft fibrous composite consisting of the UHMWPE fibers.

Methodology/ Type of Raw Material	Areal Density [g/m^2^]	Thickness [mm]	Breaking Force [kN]		Relative Elongation [%]		Tear Strength [N]	
	PN-EN ISO 2286-2:2016-11	PN-EN ISO 2286-3:2016-11	along	across	along	across	along	across
			PN-EN ISO1421:2017-02		PN-EN ISO1421:2017-02		PN-EN ISO 4674-1:2017-02	
Dyneema^®^ HB26	265 ± 1	0.37 ± 0.01	136.9 ± 2.5	120.3 ± 2.1	3.6 ± 0.2	3.2 ± 0.1	does not tear	does not tear
Dyneema^®^ HB210	136 ± 1	0.19 ± 0.01	80.80 ± 9.46	53.90 ± 1.81	4.6 ± 0.4	3.8 ± 0.4	does not tear	does not tear
Dyneema^®^ HB212	136 ± 1	0.17 ± 0.02	71.40 ± 2.81	77.8 ± 2.7	3.8 ± 0.4	3.8 ± 0.4	does not tear	does not tear
Twaron^®^ CT736	407 ± 2	0.57 ± 0.02	144.00 ± 4.64	134.00 ± 3.41	7.0 ± 0.2	6.6 ± 0.2	does not tear	does not tear

**Table 2:** Main properties of nonwovens for Fiber Reinforced Plastics T1790 C.

Methodology/ Type of Raw Material	Areal Density [g/m^2^]	Thickness [mm]	Breaking Force [kN]		Relative Elongation [%]	
	PN-EN ISO 2286-2:2016-11	PN-EN ISO 2286-3:2016-11	along	across	along	across
			PN-EN ISO1421:2017-02		PN-EN ISO1421:2017-02	
Reinforced Plastics T1790 C	30.0 ± 0.2	0.30 ± 0.02	20.5 ± 2.0	11.5 ± 2.0	30.0 ± 0.2	0.30 ± 0.02
Carbon Fabric CBXS 400	409.0 ± 0.2	0.42 ± 0.02	24 ± 6	26.5 ± 6.0	409.0 ± 0.2	0.42 ± 0.02

**Table 3:** HBP variants (two-components hybrid). Arrangement of material layers in the direction from the user’s body is as follows: non-impregnated, p-aramid woven fabric: Twaron^®^ CT736/connecting binder—thermoadhesive film → soft UHMWPE fibrous composite: Dyneema^®^ HB212, HB210 or HB26.

Raw Material/HBP	H1	H2	H3	H4	H5	H6	H7	H8	H9	H10
Twaron^®^ CT736/connecting binder—thermoadhesive film	26	22	18	26	22	18	26	22	18	18
Dyneema^®^ HB210	50	60	70	0	0	0	0	0	0	0
Dyneema^®^ HB212	0	0	0	50	60	70	0	0	0	32
Dyneema^®^ HB26	0	0	0	0	0	0	50	60	70	26
Total number of layers in the HBP	76	82	88	76	82	88	76	82	88	76

**Table 4:** HBP (three-components hybrid) materials used in both Variants A and B.

Raw Material/HBP	H4/S	H5/S	H6/S	H4/W	H5/W	H6/W
Twaron^®^ CT736/connecting binder—thermoadhesive film	26	22	18	26	22	18
Dyneema^®^ HB212	50	60	70	50	60	70
Nonwovens for Fiber Reinforced Plastics T1780 C	4	4	4	0	0	0
Carbon Fabric CBXS 400	0	0	0	2	2	2
Total number of layers in the HBP	80	86	92	78	84	90

**Table 5:** Validity of the groups of properties of HBP being the criteria for the selection of the optimal HBP variant.

Properties Groups	Feature	Validity [*t_i_*] ^1^
Physical properties	Areal density	5
	Thickness	2
Functional properties	Average velocity	5
	Average depth of the deformation	1
	Minimal depth of the deformation	5
	Deformation area	5
	Deformation volume	3
	Ballistic penetration	5

^1^ Validity: from 1—the least important feature, to 5—the most important feature.

**Table 6:** Parameters of the designed HBP selected in two groups: physical properties and usable properties.

HBP Variant	Areal Density [kg/m^2^]	Thickness [mm]	Average Velocity [m/s]	Average Depth of the Deformation [mm]	Minimal Depth of the Deformation [mm]	Deformation Area [cm^2^]	Deformation Volume [cm^3^]	Ballistic Penetration (1-No; 0-Yes)
	Physical Properties Group		Usable Properties Group					
H1	19.10	16.50	564.40	21.30	29.90	29.30	44.74	0
H2	18.60	17.00	583.70	21.70	34.60	34.17	56.83	0
H3	18.20	17.50	621.60	27.50	41.20	40.88	85.87	0
H4	18.90	16.50	605.10	21.50	28.30	32.48	52.60	0
H5	18.30	17.00	601.70	23.90	31.00	34.27	62.65	1
H6	17.70	17.50	597.60	22.10	31.00	31.30	55.32	1
H7	25.20	23.00	594.70	19.90	27.60	31.13	55.32	1
H8	26.20	24.50	589.20	19.20	26.50	29.67	48.57	1
H9	26.90	25.50	589.00	19.80	26.50	28.90	43.80	1
H10	19.40	19.00	593.50	19.30	28.70	29.98	45.53	0
H4/SA	19.10	16.00	593.60	24.10	32.00	40.32	71.92	0
H5/SA	18.60	16.50	583.60	21.90	29.20	34.00	54.77	1
H6/SA	17.90	16.50	579.90	20.00	28.20	28.78	45.15	1
H4/WA	19.50	17.00	581.90	18.10	25.70	24.70	36.12	0
H5/WA	18.90	17.00	565.30	21.10	31.40	31.03	50.57	1
H6/WA	18.30	17.00	574.30	20.80	31.10	30.00	49.53	1
H4/SB	17.90	15.00	585.35	11.00	12.00	25.17	31.03	0
H5/SB	18.50	16.50	569.67	25.57	34.90	34.47	54.52	1
H6/SB	17.90	16.50	572.43	22.85	33.10	29.67	46.13	1
H4/WB	19.40	16.50	572.15	19.44	30.50	24.90	46.13	0
H5/WB	19.00	17.00	574.08	19.93	29.50	30.90	37.18	1
H6/WB	18.5	16.5	567.00	22.25	32.70	30.48	49.57	1

**Table 7:** Selection of optimal variants of the two-components HBP.

HBP Variant	Physical Properties Group		Usable Properties Group		General Coefficient of Quality (GSQ)	General Quality Class (GQC)
	SCQ_Ph_^1^ Factor	Quality Class (C_Ph_) ^2^ Factor	SCQ_U_^1^ Factor	Quality Class (C_U_) ^2^ Factor		
H1	0.89	1	0.32	6	0.61	3
H2	0.91	0	0.37	6	0.64	3
H3	0.93	0	0.42	5	0.67	3
H4	0.91	0	0.52	4	0.71	2
H5	0.94	0	0.67	3	0.80	2
H6	0.97	0	0.63	3	0.80	2
H7	0.21	7	0.68	3	0.45	5
H8	0.09	9	0.67	3	0.38	6
H9	0.00	9	0.67	3	0.33	6
H10	0.79	2	0.46	5	0.63	3

^1^ SCQs of variants ranged from 0 to 1, where 1—perfect quality; ^2^ C = 0—ideal variant and C = 9—the most unfavorable.

**Table 8:** Selection of the optimal three-components HBP.

HBP Variant	Physical Properties Group		Usable Properties Group		General Coefficient of Quality (GSQ)	General Quality Class (GQC)
	SCQ_Ph_^1^ Factor	Quality Class (C_Ph_) ^2^ Factor	SCQ_U_^1^ Factor	Quality Class (C_U_) ^2^ Factor		
H4/SA	0.86	1	0.41	5	0.64	3
H5/SA	0.89	1	0.57	4	0.73	2
H6/SA	0.94	0	0.52	4	0.73	2
H4/WA	0.81	1	0.31	6	0.56	4
H5/WA	0.85	1	0.46	5	0.66	3
H6/WA	0.90	1	0.48	5	0.69	3
H4/SB	0.98	0	0.46	5	0.72	2
H5/SB	0.90	1	0.47	5	0.69	3
H6/SB	0.94	0	0.46	5	0.70	3
H4/WB	0.83	1	0.22	7	0.52	4
H5/WB	0.84	1	0.54	4	0.69	3
H6/WB	0.90	1	0.45	5	0.67	3

^1^ SCQs of variants ranged from 0 to 1, where 1—perfect quality; ^2^ C = 0—ideal variant; and C = 9—the most unfavorable.

## Data Availability

Not applicable.
